# Protein targets in the red complex organisms binding with an herbal compound silymarin

**DOI:** 10.6026/97320630016759

**Published:** 2020-10-31

**Authors:** Nadhirah Faiz, Keshaav Krishnaa Pothapur, V Vishnupriya, R Gayathri, J Vijayashree Priyadharsini

**Affiliations:** 1 Saveetha Dental College and Hospitals, Saveetha Institute of Medical & Technical Sciences (SIMATS), Saveetha University Chennai 600077, Tamilnadu, India

**Keywords:** Protein targets, organisms, silymarin

## Abstract

Periodontitis is attributed to the dental biofilm formation caused by various microbial changes that occurs in the biofilm. Red complex organisms are a group of organisms linked with periodontal diseases. Therefore, it is of interest to identify potential targets from the red complex organisms to bind with the herbal compound silymarin. We report a list of potential proteins having optimal drug like binding features with the herbal agent silymarin for further consideration. We used the STITCH v.5 pipeline using VICMPred and VirulentPred tools to identify such targets as potential virulent factors in the red complex organisms. We considered the strains of Porphyromonas gingivalis ATCC 33277, Treponema denticola ATCC 35405 and Tannerella forsythia ATCC 43037 in the red complex pathogens for this analysis. Protein targets in the red complex organisms with optimal binding features with the herbal compound silymarin were thus identified and reported for further consideration.

## Background

The extracts of herbs have been used for decades in traditional medicine [1]. There has been an increasing interest in the study of medicinal plants and their use in different parts of the world as potent substances against various diseases [2-6]. Nearly 80% of the world's human population depends on herbal medicine in the form of traditional medicine for the primary healthcare needs according to data from the World Health Organization. The development and use of medicinal plants for therapy carry considerable economic benefits in the treatment of various diseases [7]. 25% of the medical drugs that are in usage among the population are based on herbs and their derivatives in developed nations [8].

Silymarin is a compound that is derived from Silybum marianum and has been widely used as an effective herbal medicine in hepatic disorders [9]. The prescription of silymarin is increasing due to its safety and efficacy all over the world [10]. Hepatoprotective activities, skin protection and cancer treatment using silymarin in human healthcare is known [11,12]. Data on the protective role of silymarin in prevention and treatment of oral disease such as dental caries is also known [13]. The most common oral disease is dental carries followed by periodontal disease [14]. The etiology of periodontal diseases is bacterial plaque, which causes the destruction of the gingival tissue and the destruction of periodontal attachment apparatus [15,16]. The bacterial biofilm tends to adhere and mature in the cervical portion of the clinical crown, extending into the gingival sulcus and progresses further occlusally. A qualitative change that occurs in the microbial composition of plaque is also known [17,18]. The change in the microbial colonies leads to the growth of various groups of organisms; one such group is the red complex organisms [19]. Therefore it is of interest to identify potential targets from the red complex organisms to inhibit the herbal compound Silymarin.

## Methodology

### Workflow:

It is of interest to identify potential targets from the red complex organisms to inhibit the herbal compound Silymarin. STITCH 5 [20] was used to identify potential proteins interacting with Silymarin. Their virulence properties were predicted using VICMPred [21] and VirulentPred [22]. Porphyromonas gingivalis ATCC 33277, Treponema denticola ATCC 35405, Tannerella forsythia ATCC 43037 strains of the red complex pathogens were considered in this study.

### Prediction of protein-drug interactions:

STITCH database (Version 5; 2016) is a comprehensive platform for known and predicted interactions between proteins and putative bioactive compounds. A repertoire of proteins from P. gingivalis, T. denticola, and T. forsythia, were used for predicting virulence. [20]

### Virulence prediction:

VICMpred [21] and VirulentPred [22] pipelines were used for the identification of virulence factors inhibited by Silymarin in red complex pathogens. These tools employed support vector machine [SVM]-based five-fold cross-validation process for prediction. Potential Virulence factors were predicted using VirulentPred. VICMpred categorizes proteins into four major classes, such as, proteins involved in cellular process, metabolism, information storage, and virulence. Protein sequences were retrieved from the NCBI database for this analysis [23].

### Prediction of subcellular localization of the virulent proteins:

The prediction of localisation of proteins at a sub cellular level helps in designing unique drug targets for substantiating the role of an antimicrobial agent, which targets the virulent protein. Cell surface proteins are of great interest as vaccine targets. PSORTb V3.0 is an algorithm, which assigns a probable local site to a protein from sequence data [24].

## Results and Discussion:

**Figure 1:**
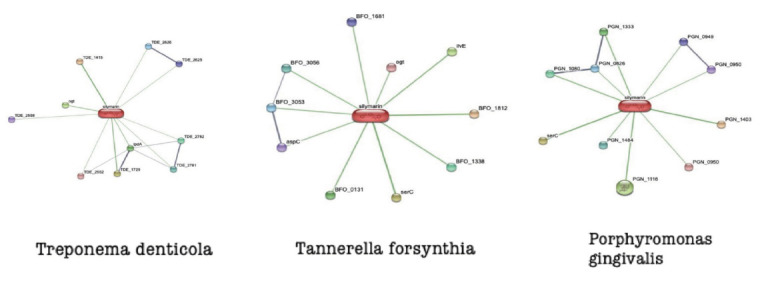
Illustration of protein targets having interaction networks in different red complex pathogens

The STITCH pipeline was used to identify the proteins having interaction from red complex bacteria with the herbal compound Silymarin (Figure 1). Each protein found interacting with the compound was assessed for their virulence property using VirulentPred andVICMpred. The scores produced by the algorithms grouped them into two classes, virulent and avirulent. Drug Protein interactions were primarily related to cellular processes in P. gingivalis, followed by metabolism and virulence factor (Table 1 - see PDF). The scores from VirulentPred marked carboxy norspermidine decarboxylase and Superoxide dismutase Fe-Mn as virulent factors. STITCH prediction for Silymarin returned proteins (Table 1 - see PDF) mainly associated with metabolism and cellular processes in T. denticola. Pyridoxyl dependent family decarboxylase and hypothetical protein, associated with metabolism and cellular process respectively were found to be virulent based on the score obtained from VirulentPred. Majority belonged to cellular Process, followed by metabolism and virulence factor in T. forsythia interacting with in silymarin. Serpin associated with metabolism and carboxy norspermidine decarboxylase were also predicted to be associated with virulence.

The establishment of an association of periodontal diseases and systemic diseases has been previously implied. A direct relation has been established between diabetes and cardiovascular diseases. A relationship between liver cirrhosis and periodontal diseases is also known [25]. It was found that there was an increased incidence of periodontitis in cases of liver cirrhosis. Hence, it is critical to study and see if the drugs utilized in the treatment of liver diseases will be helpful to treat the accompanying disease like periodontitis.

Data on the antifungal activity of silymarin with 5 reference strains of Candida is known [26]. Data presented here shows that Silymarin is of potential use in the down regulation of the virulence factors by destabilisation of the mature biofilm by inhibition of hydrolases in the local environment in the context.

## Conclusion

We report a list of potential proteins from the red complex organisms having optimal drug like binding features with the herbal agent silymarin for further consideration.
